# Massive Spontaneous Right Hemothorax Revealing Acute Myeloid Leukemia: A Case Report and Literature Review

**DOI:** 10.7759/cureus.79422

**Published:** 2025-02-21

**Authors:** Said Khallikane, Amine Bentahar, Mounir Reda, Monsef Elabdi, Monsif Salek

**Affiliations:** 1 Cardiothoracic Anesthesiology, Avicenna Military Hospital, Marrakech, MAR; 2 Medicine and Pharmacy, Cadi Ayyad University, Marrakech, MAR; 3 Diagnostic and Interventional Radiology, Moulay Ismail Military Hospital, Meknes, MAR; 4 Cardiovascular Surgery, Avicenna Military Hospital, Marrakech, MAR; 5 Traumatology and Orthopedics, Hassan II Military Hospital, Laayoune, MAR; 6 Medicine, Pharmacy, and Dentistry, Sidi Mohamed Ben Abdellah University, Meknes, MAR

**Keywords:** acute myeloid leukemia, coagulopathy, leukostasis, spontaneous hemopericardium multidisciplinary approach, spontaneous hemothorax, thrombocytopenia, tube thoracostomy

## Abstract

Acute myeloid leukemia (AML) is a malignant hematologic disorder characterized by bone marrow failure and an increased risk of hemorrhagic complications due to thrombocytopenia, coagulopathy, and hyperfibrinolysis. While AML is associated with bleeding tendencies, massive spontaneous hemothorax (SH) is an exceedingly rare and often fatal manifestation. We report the case of a patient who presented with acute chest pain, dyspnea, and hemodynamic instability, ultimately diagnosed with AML-associated hemothorax. Imaging confirmed a large pleural effusion, and pleural fluid analysis revealed a hematocrit consistent with hemothorax. Bone marrow biopsy established a diagnosis of acute myelomonocytic leukemia (AML M4, FAB classification) with myelodysplasia-related changes (WHO classification). Management included urgent tube thoracostomy, correction of coagulopathy, and fluid resuscitation, with surgical intervention considered based on bleeding progression. Despite supportive measures, the prognosis in AML-associated hemothorax remains poor due to the underlying malignancy and hemorrhagic complications. During disease progression, the development of spontaneous hemopericardium further worsened the prognosis, leading to severe hemodynamic deterioration and increased mortality risk. This case underscores the importance of early recognition, rapid diagnosis via ultrasound and CT imaging, and a multidisciplinary treatment approach involving hematologists, intensivists, and thoracic specialists. Given the rarity of this complication and the absence of standardized guidelines, further studies are needed to refine diagnostic and therapeutic strategies for AML-related hemothorax.

## Introduction

Acute myeloid leukemia (AML) is a malignant clonal disorder characterized by the uncontrolled proliferation and differentiation arrest of immature myeloid progenitor cells in the bone marrow. While AML is commonly associated with bleeding tendencies due to thrombocytopenia and coagulation abnormalities, massive spontaneous hemothorax (SH) is an exceptionally rare and life-threatening complication, often unrecognized in its early stages. Although AML frequently presents with pleural effusion, pulmonary infiltrates, and pleurisy, hemothorax remains an unusual manifestation with high mortality [1,2].

SH in AML has been reported primarily in patients with post-chemotherapy marrow aplasia, whereas significant bone marrow involvement has been less frequently observed. The role of leukemic infiltration in severe pulmonary complications, particularly vascular invasion and epithelial proliferation, remains unclear [1,3,4]. The proposed mechanisms include coagulopathy, leukemic leukostasis, and alveolar infiltration leading to vascular fragility and hemorrhage. In rare instances, fibro-lamellar tissue formation in the thoracic cavity has been linked to diaphragmatic rupture and pseudo-chylothorax [1,3].

In addition to hemothorax, spontaneous hemopericardium can further complicate AML cases, as observed in our patient. The development of hemopericardium following hemothorax significantly worsened the prognosis, leading to severe hemodynamic compromise and the eventual progression to cardiac tamponade. Given its rapid deterioration, early recognition through echocardiography and prompt management with pericardiocentesis are crucial in preventing fatal outcomes.

This case highlights the critical importance of early recognition and intervention in AML-related SH, particularly in coagulopathic patients. Given its rarity and poor prognosis, a timely multidisciplinary evaluation is essential to improve outcomes. Further studies are needed to better understand its pathophysiology, refine treatment strategies, and establish evidence-based management guidelines.

## Case presentation

A 72-year-old man with no prior hematologic history was admitted with progressive shortness of breath, cough, and atypical right-sided chest pain evolving over several days. Upon admission, he was in evident respiratory distress, with sweating, rapid shallow breathing, and tachypnea at 25 breaths per minute with a pulse oxygen saturation (SpO_2_) of 88% on room air, which improved to 98% under high-concentration oxygen therapy. His blood pressure was 80/54 mmHg, and his pulse was 88 bpm. On general examination, the patient presented with mucocutaneous pallor, weight loss, and purpuric lesions in the form of generalized petechiae. Respiratory examination revealed thoracic asymmetry with bulging of the right hemithorax, without tympanism on percussion. Additionally, pulmonary auscultation showed muffled breath sounds and reduced chest expansion in the right hemithorax, with no clinical signs of heart failure, and non-palpable organomegaly was observed. The lymph node regions were non-palpable and without lymphadenopathy. The examination of the upper airways revealed extensive oral thrush, along with pseudomembranous pharyngitis presenting as whitish plaques, suggestive of oropharyngeal candidiasis. The abdomen was tender, particularly in the right hypochondrium, without clear signs of peritoneal defense. Bladder catheterization showed clear urine. The rest of the clinical examination was unremarkable.

Laboratory findings at admission

The initial laboratory workup revealed severe pancytopenia, with anemia (Hb: 5.9 g/dL), profound thrombocytopenia (14×10³/μL), and marked leukocytosis (38.7×10³/μL), including a neutrophil count of 12×10³/μL. Despite the leukocytosis, the patient exhibited mildly reduced lymphocyte count (850/µL). Coagulation studies revealed a prolonged prothrombin time (PT: 40.7%) and a mildly elevated activated partial thromboplastin time (aPTT, TCA: 50 sec). Factor V levels were normal (75%), while platelet aggregation testing by photometry was significantly impaired, and fibrinogen levels were critically low (1 g/L). Additionally, D-dimer levels were markedly elevated at 15,000 ng/mL, strongly suggesting a consumptive coagulopathy.

The hepatic panel was within normal limits, with AST 35 IU/L, ALT 32 IU/L, GGT 55 IU/L, ALP 80 IU/L, and total bilirubin 15 µmol/L (direct bilirubin 0.6 mg/dL). Phosphocalcic balance was preserved, with normal parathyroid hormone (PTH: 45 pg/mL), calcium, phosphate, and electrolyte levels (sodium, potassium, and chloride). The endocrine function was unremarkable, with morning cortisol at 60 µg/dL, a normal ACTH stimulation test (Synacthen peak cortisol: 34 µg/dL), and normal thyroid function (TSH within range). In contrast, renal function was impaired, with elevated urea (12 mmol/L) and creatinine (150 µmol/L), corresponding to an estimated glomerular filtration rate (eGFR) of ~40 mL/min/1.73 m² (MDRD equation) and a creatinine clearance of ~36.7 mL/min (Cockcroft-Gault formula), indicating moderate renal insufficiency. Additionally, uric acid was slightly elevated, and ferritin levels were significantly increased. Cardiac biomarkers were abnormal, with elevated troponin I and NT-proBNP, raising concern for myocardial strain. The C-reactive protein (CRP) level was 445 mg/L, and procalcitonin (PCT) was 4 ng/mL (Table 1).

**Table 1:** Laboratory findings.

Parameter	Value	Normal range
Hemoglobin (Hb)	5.9 g/dL	13.5-17.5 g/dL (M), 12.0-15.5 g/dL (F)
Platelet count	14×10³/μL	150-450×10³/μL
White blood cell count (WBC)	38.7×10³/μL	4.0-11.0×10³/μL
Neutrophils	12×10³/μL /µL	2000-7500/µL
Lymphocytes	850/µL	1000-4000/µL
Lymphoblasts	80%	Absent
Prothrombin time (PT)	40.7%	70-100%
Activated partial thromboplastin time (aPTT)	40 sec	25-35 sec
Factor V	75%	50-150%
Platelet aggregation	Impaired	Normal
Fibrinogen	1 g/L	2-4 g/L
D-dimer	15000 ng/mL	<500 ng/mL
AST	35 IU/L	<40 IU/L
ALT	32 IU/L	<45 IU/L
GGT	55 IU/L	10-60 IU/L
ALP	80 IU/L	40-130 IU/L
Total bilirubin	15 µmol/L	3-20 µmol/L
Direct bilirubin	0.6 mg/dL	<0.3 mg/dL
Urea	12 mmol/L	2.5-7.1 mmol/L
Creatinine	150 µmol/L	53-106 µmol/L
eGFR (MDRD)	~40 mL/min/1.73m²	>60 mL/min/1.73m²
Creatinine clearance (Cockcroft-Gault)	~36.7 mL/min	>90 mL/min
Uric acid	12 mg/dL	Male: 3.4-7.0 mg/dL, female: 2.4-6.0 mg/dL
Ferritin	700 ng/mL	30-300 ng/mL (M), 15-200 ng/mL (F)
Troponin I	50 ng/mL	<14 ng/mL
NT-proBNP	800 ng/mL	<125 pg/mL (<75 years)
C-reactive protein (CRP)	445 mg/L	<10 mg/L
Procalcitonin (PCT)	4 ng/mL	<0.05-0.5 ng/mL

Arterial blood gas (ABG) analysis revealed a mixed metabolic and respiratory acidosis with hypoxemia, consistent with respiratory failure and potential tissue hypoperfusion. The remainder of the metabolic panel was unremarkable (Table 2).

**Table 2:** ABG analysis. ABG, arterial blood gas

Parameter	Value	Normal range	Interpretation
pH	7.25	7.35-7.45	Acidemia
PaCO₂	60 mmHg	35-45 mmHg	Respiratory acidosis (hypercapnia)
PaO₂	41 mmHg	80-100 mmHg	Severe hypoxemia
HCO₃⁻ (bicarbonate)	16 mEq/L	22-26 mEq/L	Metabolic acidosis
Base excess (BE)	-9 mEq/L	-2 to +2 mEq/L	Significant base deficit (metabolic component)
Lactate	3 mmol/L	0.5-2.2 mmol/L	Elevated (likely due to hypoperfusion/shock)
Anion gap	21 mEq/L	8-16 mEq/L	High (suggests anion gap metabolic acidosis - AGMA)
Ionized calcium (Ca²⁺)	1.15 mmol/L	1.12-1.3 mmol/L	Normal

These findings suggest a complex hematologic disorder with severe coagulopathy, renal impairment, and metabolic disturbances, necessitating urgent multidisciplinary intervention. The presence of disseminated intravascular coagulation (DIC), potential tumor lysis-like syndrome, and evolving organ dysfunction highlights the severity of this case, reinforcing the need for aggressive supportive care and urgent diagnostic clarification.

**Figure 1:**
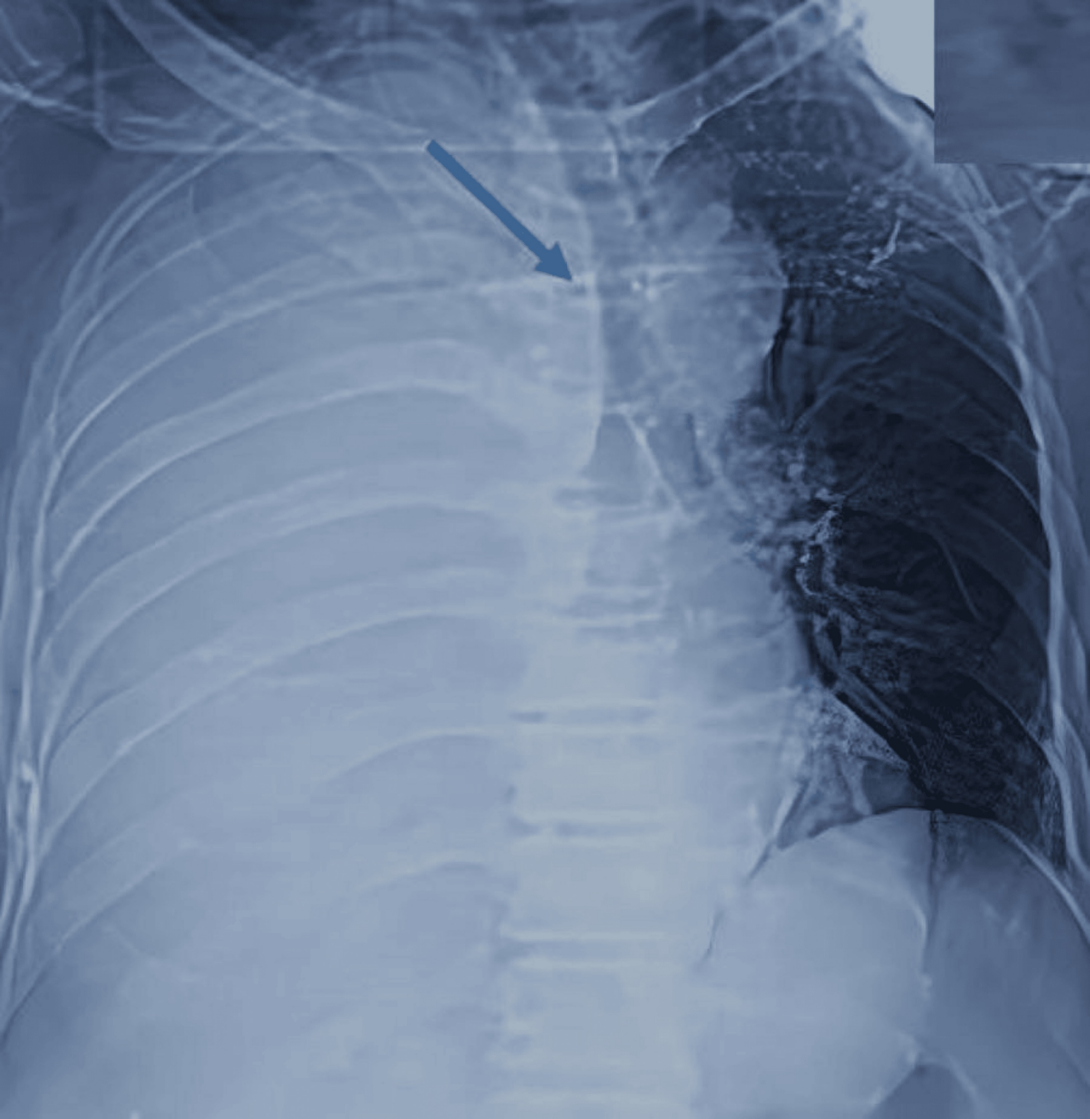
Chest X-ray in the AP view showing a white right lung with displacement of the mediastinal structures (trachea) toward the contralateral side, consistent with large-volume pleurisy. AP, anteroposterior

**Figure 2:**
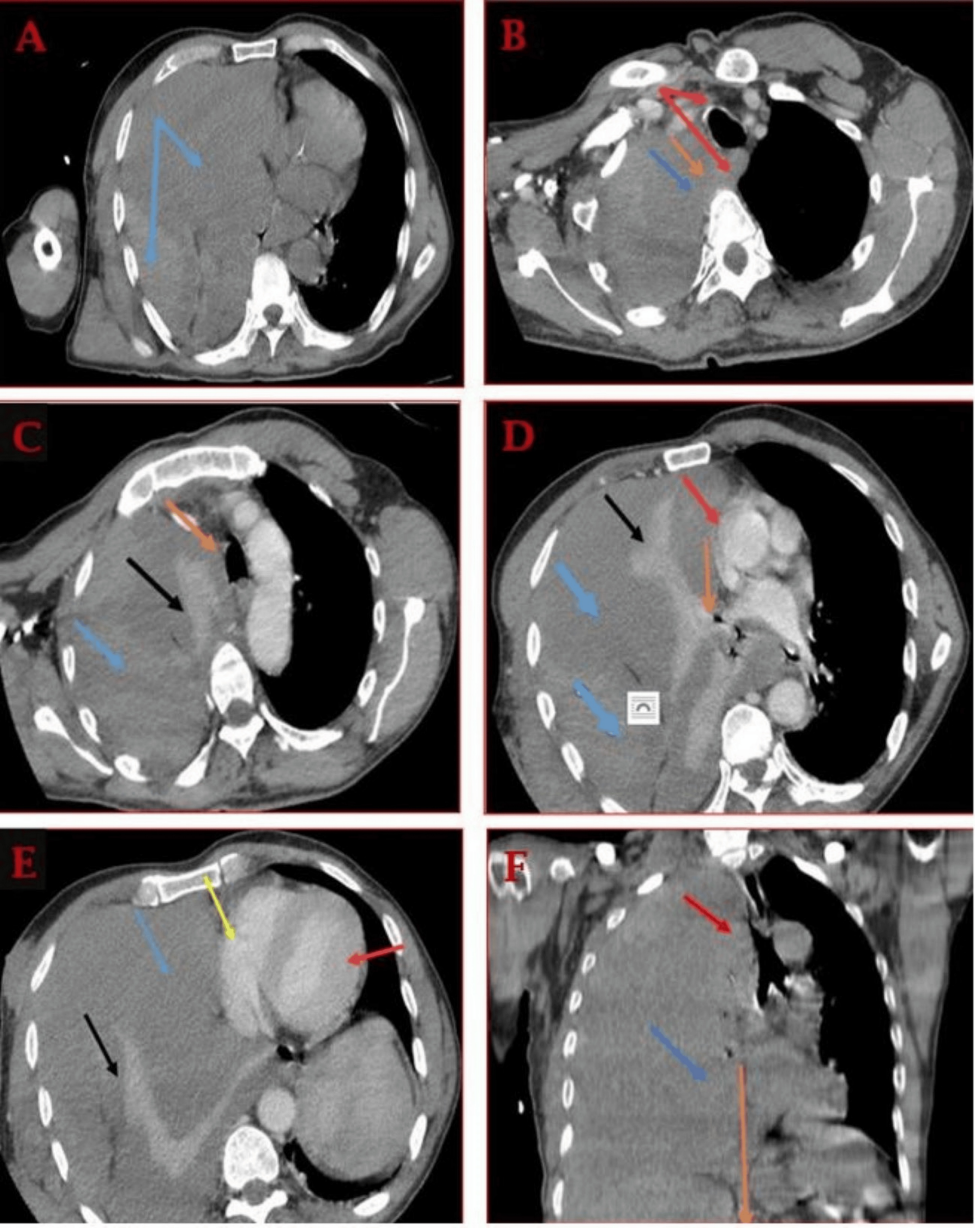
Chest CT scan before and after contrast enhancement in axial and coronal views shows a large, right heterogeneous pleural effusion with spontaneously hyperdense areas (blood) (A-E) and darker areas (fluid), creating a fluid-blood level suggestive of a massive hemothorax (blue arrows) (A-F). Possible mediastinal lymph nodes are noted in the upper mediastinum between the opacified supra-aortic trunks (B, D) and behind the trachea (B) (red arrows). Pulmonary collapse is also observed at the right pulmonary hilum (C-E) (black arrow). There is a significant mass effect exerted by the right hemothorax on the mediastinal structures (B, C, D) and heart (yellow arrow) (E), which are displaced toward the contralateral side with no visible pericardial effusion (E) (red arrow).

A posteroanterior chest X-ray revealed a white right lung with displacement of the mediastinal structures (trachea) toward the contralateral side, consistent with a large-volume pleural effusion (Figure 1). This was confirmed by both non-contrast and contrast-enhanced chest CT, which showed the presence of a substantial, heterogeneous pleural effusion, characterized by spontaneously hyperdense regions of blood interspersed with darker fluid areas. This indicates the formation of a fluid-blood interface, suggestive of a massive hemothorax. Additionally, potential mediastinal lymph nodes are identified in the upper mediastinum, located between the opacified supra-aortic trunks and posterior to the trachea. Furthermore, there is evidence of pulmonary collapse at the right pulmonary hilum. The substantial hemothorax imposes a considerable mass effect on the mediastinal structures and the heart, leading to displacement toward the opposite side, while no pericardial effusion is discernible, and no focal traumatic defect is observed (Figure 2).

**Figure 3:**
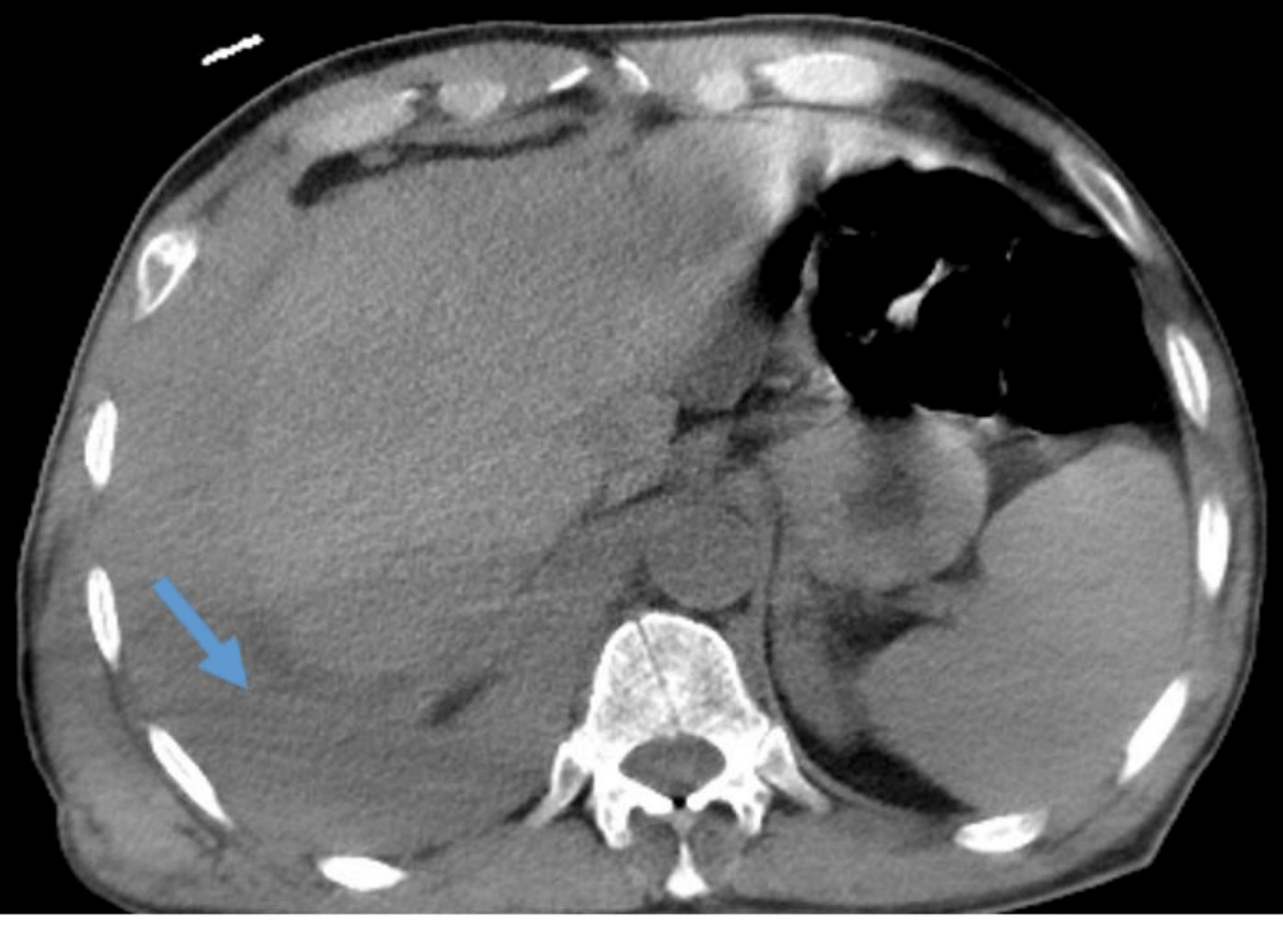
A non-contrast-enhanced abdominal CT scan reveals perihepatic effusion and fluid accumulation in the supramesocolic compartment.

Additionally, extended CT scan slices at the upper abdominal level revealed a non-contrast abdominal CT scan that shows perihepatic effusion and fluid accumulation in the supramesocolic compartment (Figure 3).

Repeated aerobic and anaerobic blood cultures were negative; however, a swab from the oral thrush confirmed the presence of Candida albicans sensitive to fluconazole. A cytobacteriological respiratory multiplex panel on oropharyngeal aspirations and sputum tested positive for Moraxella catarrhalis and cytomegalovirus (CMV), while urine cytobacteriological examination was negative. Serologic testing revealed CMV IgM positivity, indicating a recent or active infection, with PCR for CMV DNA positive in both blood and gingival biopsy tissue. Other viral serologies, including HIV, were negative. Pleural cytology identified macrophages with hemosiderin deposits and red blood cell aggregates, with no malignant cells, confirming a hemorrhagic effusion. Additionally, bone marrow biopsy confirmed acute myelomonocytic leukemia (AML Muc4, FAB classification) with myelodysplasia-related changes (WHO classification).

Treatment evolution and outcome

The patient received intravenous fluids and norepinephrine at 1.5 µg/kg/min, along with a massive transfusion protocol, including 10 packed red blood cells (PRBCs), 10 fresh frozen plasmas (FFPs), and two platelet concentrates (PCs) (1.2×10¹¹ per 10 kg of body weight) to correct thrombocytopenia and control active bleeding. Fibrinogen replacement was initiated with 2 g of fibrinogen concentrate IV, and tranexamic acid (Exacyl®) was administered via continuous infusion, starting with an initial bolus of 2 g IV over one hour, followed by 1 g after three hours, and a maintenance infusion at 1 mg/kg/h over eight hours. Subsequent fibrinogen level monitoring indicated the need for an additional 1 g of fibrinogen concentrate to achieve a target fibrinogen level of ≥2 g/L, leading to hemodynamic stabilization and adequate diuresis.

**Figure 4:**
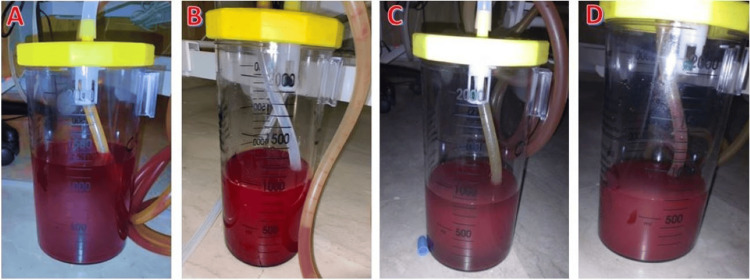
The images represent the progressive evacuation of a massive hemothorax via echo-guided drainage through the 3rd to 5th intercostal space. The collected fluid volume and hematocrit (>50%) indicate a significant acute hemorrhagic pleural effusion. Initial drainage of 1300 mL of hematic fluid, which appears dark red and homogeneous, confirms a large hemothorax. The drainage was well-positioned, with efficient evacuation of the pleural cavity (A). After clamping and declamping of the chest drain, as the hemorrhage initially appeared active, the subsequent drainage yielded 1000 mL of blood. The decrease in volume suggests partial lung re-expansion and reduced thoracic compression, while the high-hematocrit blood confirms ongoing pleural evacuation (B). The third drainage phase evacuated 900 mL, indicating progressive resolution of the hemothorax. The suction system remained functional, with no signs of clot formation or obstruction observed. Furthermore, the patient likely experienced improved respiratory mechanics at this stage (C). The final drainage phase evacuated 700 mL, marking a significant reduction in intrapleural blood volume. The lower collected volume suggests diminished active bleeding, while massive transfusion was maintained, contributing to the hemodynamic and respiratory stabilization of the patient (D).

**Figure 5:**
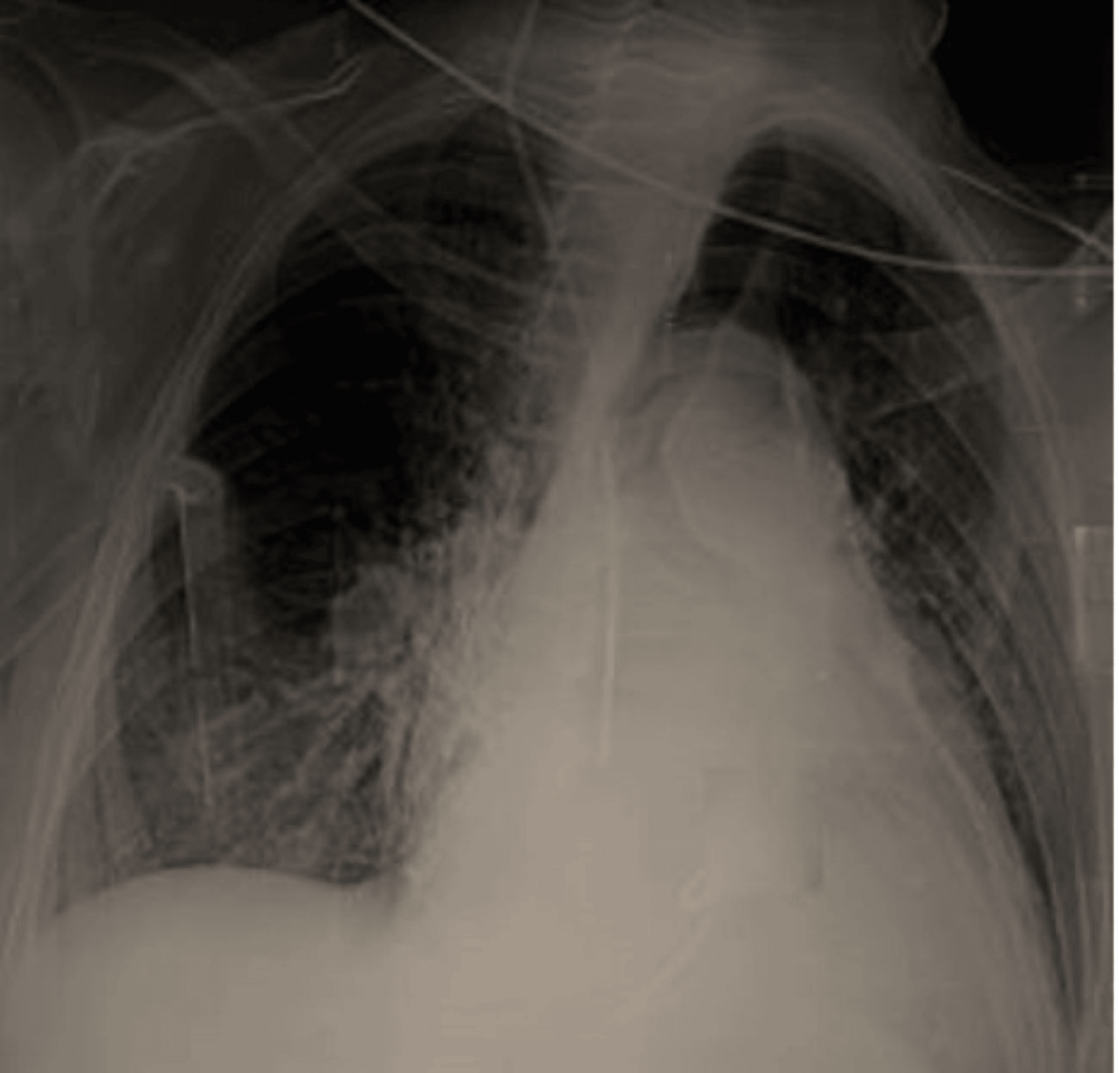
AP chest X-ray of the same patient after drainage, showing a complete resolution of the effusion. AP, anteroposterior

Additionally, thoracic drainage of 1 L of blood further improved stabilization. A 36 Fr mediastinal chest tube was inserted, initially draining 1700 mL of blood, with a hematocrit of 55%, confirming a massive hemothorax. This was followed by an additional 1 L, then 800 mL over four hours, and 700 mL before gradually tapering off (Figure 4 and Figure 5).

The hemostatic assessment revealed hyperfibrinolysis. Given the presence of neutropenia and significantly elevated infectious markers, broad-spectrum antimicrobial therapy was initiated, despite Moraxella catarrhalis being the only isolated pathogen, which was sensitive to amoxicillin and ceftriaxone. The patient was started on intravenous Ceftazidime at 6 g per 24 hours via an electric syringe pump, Moxifloxacin 400 mg every 12 hours, Amikacin 1.5 g per day, and Fluconazole with a loading dose of 400 mg every 12 hours, followed by 200 mg every 12 hours, along with Miconazole oral gel. Additionally, oral hygiene was maintained with a mouthwash containing chlorhexidine digluconate (0.1%) and chlorobutanol (0.5%).

A follow-up assessment was performed, and after achieving infection control following seven days of antimicrobial treatment and normalization of PCT, emergency chemotherapy was initiated on the 8th day of admission due to persistent hyperleukocytosis with circulating lymphoblasts. The assessment after induction chemotherapy is shown in Table 3.

**Table 3:** Post-chemotherapy laboratory findings.

Parameters	Value	Reference range
Hemoglobin	5.9 g/dL (9.9 g/dL g/dL later)	13-17 g/dL
White blood cells	38.7×10³ (11.77×10³/μL/L later)	4.0-11.0×10⁹/L
Lymphoblasts	80% (5%)	Absent
Platelets	14×10³/μL (↑70 G/L later)	150-450×10⁹/L
Prothrombin time	40.7%→47%	>70%
INR	1.24	<1.2
D-dimer	14,480 μg/L	<500 μg/L
Fibrinogen	2.5 g/L	2.0-4.0 g/L
Lactate	3.5 mmol/L→1.60 mmol/L	<2.0 mmol/L
Creatinine	150 µmol/L→124 μmol/L	53-115 μmol/L
NT-proBNP	800 ng/mL→552 ng/mL	
Troponin I	50 ng/mL→102 ng/mL	<14 ng/mL
C-reactive protein (CRP)	445 mg/L→50 mg/L	<10 mg/L
Procalcitonin (PCT)	4 ng/mL→0.75 ng/mL	<0.05-0.5 ng/mL

**Figure 6:**
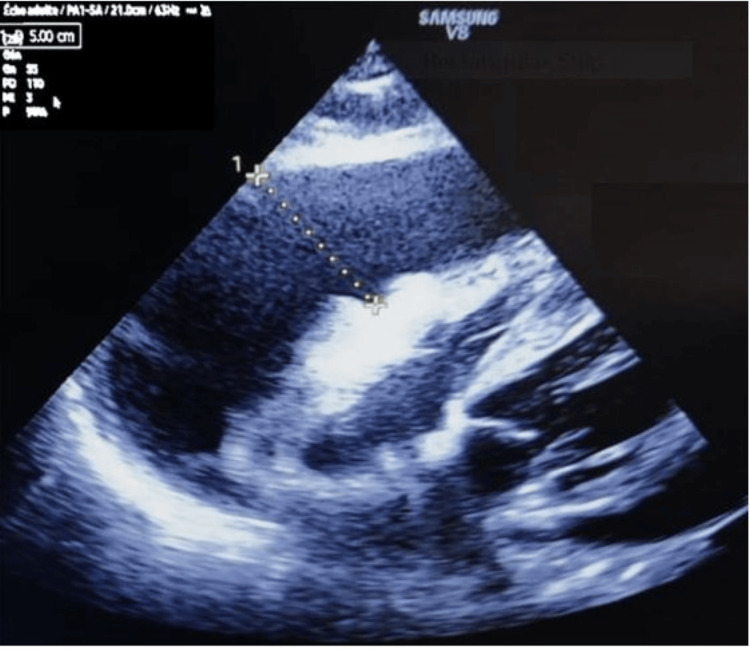
The subxiphoid (subcostal) TTE view shows a large pericardial effusion, evidenced by a significant anechoic fluid collection surrounding the heart. The measured effusion thickness is substantial, confirming severe pericardial fluid accumulation. TTE, transthoracic echocardiographic

**Figure 7:**
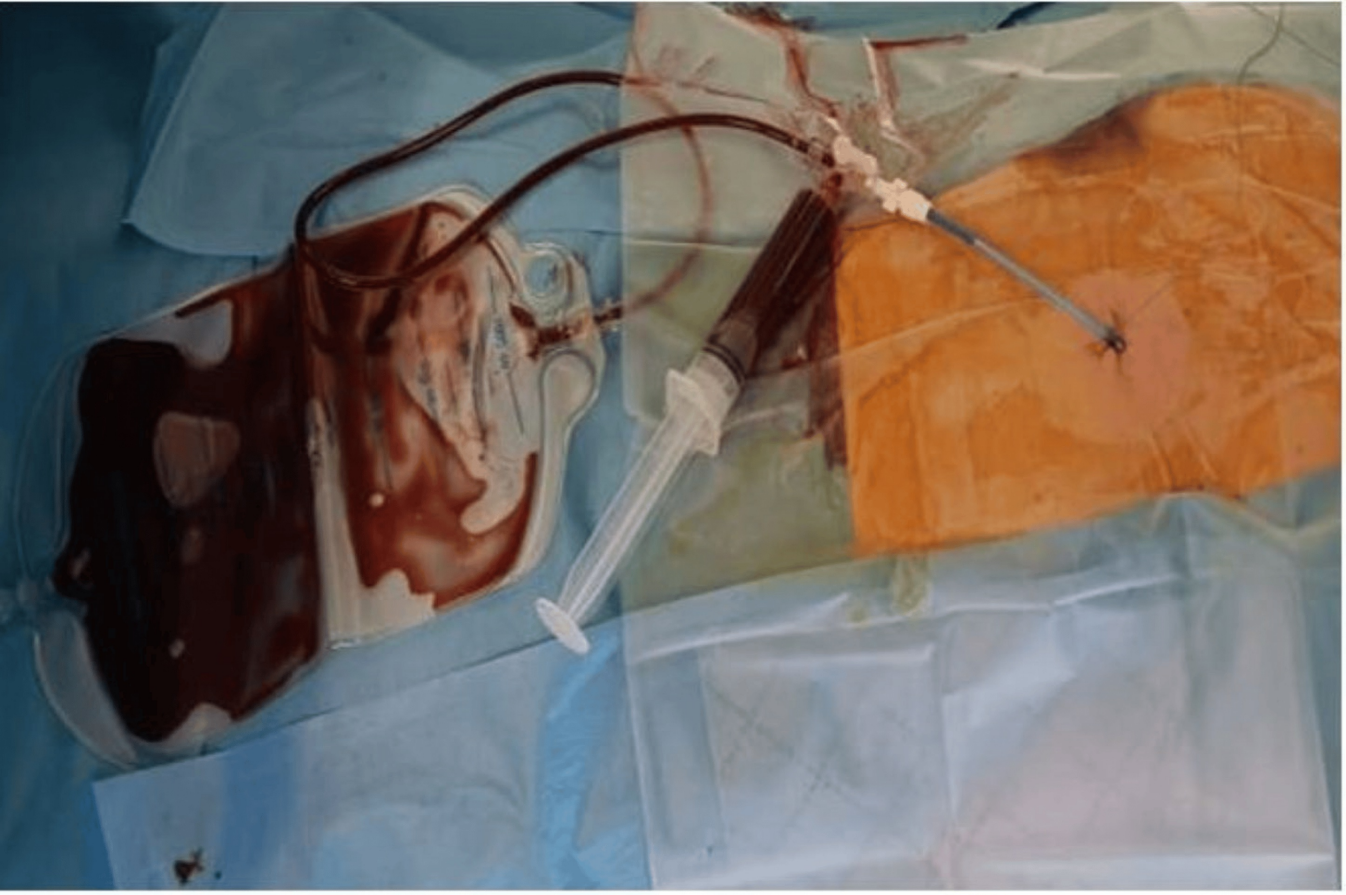
The image illustrates a subxiphoid pericardial drainage procedure performed for a large spontaneous hemopericardium. A pericardial catheter is in place, facilitating the evacuation of a significant volume of hemorrhagic pericardial effusion into a drainage bag. The presence of dark blood confirms the hemorrhagic nature of the effusion.

Despite this intensive management, on day 12, the patient developed severe hemodynamic instability, presenting with dyspnea, orthopnea, desaturation, and signs of heart failure, including jugular vein distension, pulsus paradoxus, tachycardia with muffled heart sounds, and bibasilar crackles on pulmonary auscultation. ECG showed supraventricular tachycardia (110 bpm), with extreme right axis deviation, nonspecific ST-segment depression, and diffuse T-wave inversions, but no signs of acute ischemia, right ventricular strain, or left ventricular overload. Given the low peripheral perfusion, a differential diagnosis of massive pulmonary embolism or intrathoracic cardiac tamponade was considered. High-sensitivity troponin I (>200 ng/L) was markedly elevated, and transthoracic echocardiography confirmed a large circumferential pericardial effusion, with right ventricular diastolic collapse, right atrial compression, and swinging heart motion, strongly suggesting cardiac tamponade (Video 1) (Figure 6). Following stabilization with norepinephrine, cautious fluid resuscitation, and semi-seated positioning, the patient underwent ultrasound-guided subxiphoid pericardiocentesis, leading to hemodynamic and respiratory improvement (Figure 7). A comprehensive coagulation workup, infectious panel, and organ dysfunction assessment were performed, and pericardial fluid was sent for cytobacteriological, fungal, and neoplastic cell analysis.

**Video 1:** The subxiphoid (subcostal) TTE view shows a large pericardial effusion, evidenced by a significant anechoic fluid collection surrounding the heart. The effusion is circumferential, extending around both ventricles. The size of the effusion was measured, confirming a severe pericardial effusion. The right ventricle collapses during diastole, with swinging heart movement, and the right atrium appears compressed. These findings are hallmark signs of a large effusion, suggesting cardiac tamponade. TTE, transthoracic echocardiographic

However, the clinical course rapidly deteriorated with the onset of septic shock of pulmonary origin, leading to multiorgan failure, including DIC. The patient was intubated, mechanically ventilated, and placed on high-dose norepinephrine, dobutamine, and epinephrine for circulatory support, requiring 15 units of platelet transfusions, FEIBA at 75 U/kg IV every eight hours, 10 PRBCs, and 2 g of fibrinogen concentrate to manage coagulopathy. Broad-spectrum antimicrobial therapy was initiated with meropenem, amikacin, tigecycline, and voriconazole, following the rapid collection of microbiological samples, including aerobic and anaerobic blood cultures, fungal cultures, pericardial and pleural fluid cultures, urine cultures and cytology (UCC), cytobacteriological analysis, fungal DNA PCR (Candida PCR), and Multiplex PCR of sputum culture and cytology, tracheal aspirate cultures (TAC), bronchoalveolar lavage (BAL), and colonization index testing after catheter exchange and urinary catheter removal. A comprehensive infectious workup was conducted, including beta-D-glucan (BDG), fungal DNA PCR (Candida PCR), and multiplex PCR studies on blood and urine samples.

Cytobacteriological analysis and multiplex testing of BAL fluid identified Klebsiella pneumoniae and a multidrug-resistant Acinetobacter strain resistant to broad-spectrum beta-lactams (including carbapenems and monobactams) but sensitive only to colistin, along with pulmonary aspergillosis. Despite aggressive antifungal and antibacterial therapy, including the initiation of colistin at a dose of 2.5 MIU every 12 hours, adjusted according to the glomerular filtration rate due to worsening renal function, and caspofungin with a loading dose of 70 mg followed by a maintenance dose of 50 mg per day, the patient's condition worsened. He developed severe metabolic acidosis and anuria for over 36 hours, refractory to optimized fluid resuscitation and high-dose furosemide (1 g/24h via continuous infusion pump), necessitating emergent dialysis.

Despite intensive supportive care, hematopoietic growth factors for neutropenia, and aggressive antifungal and antibacterial therapy, the clinical course remained refractory, and the patient succumbed on day 21 of admission.

This case highlights SH as a rare and life-threatening initial presentation of AML, emphasizing the need for early recognition of hematologic malignancies in cases of massive pleural hemorrhage. Moreover, it underscores the critical challenges in managing hematologic emergencies, including determining the optimal timing for chemotherapy initiation, recognizing and preventing recurrent hemorrhage, and addressing severe infectious complications in immunocompromised patients. The poor prognosis associated with this condition, as widely reported in the literature, is consistent with the unfavorable course observed in this patient, reinforcing the urgent need for a multidisciplinary approach to optimize outcomes in these complex cases.

## Discussion

Hemothorax is typically caused by trauma, coagulopathy, or iatrogenic factors (e.g., central line placement, thoracentesis, or pleural biopsy). It is diagnosed by pleural fluid hematocrit >50% of total blood volume, though dilution in chronic cases may lower this threshold to 25-50%, mimicking hemorrhagic exudate. Pneumothorax (PTX) is one of the most common causes of hemothorax, occurring in approximately 5% of cases due to shearing forces on pleural adhesions. The lack of lung tamponade allows systemic pressure-driven bleeding, which exceeds pulmonary arterial pressure, leading to blood accumulation in the pleural space [5].

SH occurs without trauma and can present with acute chest pain, dyspnea, and hemodynamic instability, potentially leading to hypovolemic shock as was the case with our patient. It is rare, with reports primarily limited to case studies [4], while hemothorax is a rare but life-threatening complication of AML, primarily driven by thrombocytopenia, hyperfibrinolysis, and leukemic infiltration of the pulmonary vasculature [1,2]. Key mechanisms include endothelial damage from leukostasis, fragile neovessels due to angiogenesis, and hyperviscosity-related coagulopathy, all of which significantly increase the bleeding risk [3]. In some cases, hemothorax may be the initial manifestation of AML, as seen in an elderly patient with a spontaneous 2,700 mL left hemothorax, severe coagulopathy (prothrombin index 33%), and generalized symptoms. Emergent chest X-ray, chest CT, and/or chest ultrasound confirmed free fluid, prompting tube thoracostomy. Managing AML-associated hemothorax requires a multidisciplinary approach involving hematologists, intensivists, and thoracic specialists. Rapid diagnosis via chest ultrasound is crucial, followed by tube thoracostomy to evacuate the hemothorax and relieve respiratory distress. In cases of severe thrombocytopenia, platelet transfusion before invasive procedures is necessary to minimize bleeding risks [2,6]. AML is a clonal hematopoietic malignancy that predisposes patients to infections due to bone marrow failure, immunosuppression, and neutropenia, along with an increased risk of anemia and bleeding. Common in older adults, it carries a high fatality rate and complications like coagulopathies, including hemothorax. Advances in diagnostics and targeted therapies have transformed management, emphasizing personalized treatment based on patient goals, comorbidities, and genetic profiles [7,8]. The management approach depends on patient stability. Thoracoscopic or thoracostomy tube drainage of the hemothorax and re-expansion of the lung is typically used for stable patients, in addition to fluid resuscitation and blood transfusion, this was the approach used for our patient. For hemodynamically unstable patients or those with a bleeding rate exceeding 500 mL/hr in the first hour, followed by 200-300 mL/hr subsequently, an early surgical approach is favored. Correction of coagulopathy is mandatory in every case of anticoagulant-induced bleeding. Embolization remains a valid option for treating vascular abnormalities. The management of residual hemothorax after chest drain insertion remains controversial. Some patients may be potential candidates for surgery, either through early video-assisted thoracoscopic surgery (VATS), with a growing number favoring this minimally invasive method, or open thoracotomy, especially if significant clot formation is present to prevent fibrothorax and restrictive physiology [9]. A retrospective study of 24 patients with spontaneous hemopneumothorax (SHP) compared two treatment approaches: 11 patients who underwent early VATS and 13 who received initial conservative management followed by surgery if needed. The results showed that the early VATS group had longer operating times but experienced less preoperative blood loss, fewer transfusions, shorter chest tube drainage, and a shorter hospital stay. Early VATS should be considered in SHP management, as it leads to fewer postoperative complications and shorter recovery times compared to open thoracotomy [5]. In the presence of a bloody effusion, the first step is to check the hematocrit to confirm a hemothorax, as stated above, with a value between 25% to 50%, which was found in our patient. It is important to consider that a hemothorax can appear as a hemorrhagic effusion with a lower hematocrit due to significant dilution over three to four days. An enhanced CT of the chest can also provide helpful information about the etiology. For neoplastic etiologies, cytology of the pleural fluid is useful, but immunochemical markers may increase the yield and degree of confidence. In our case, pleural fluid cytology revealed macrophages with hemosiderin deposits and red blood cell aggregates but no malignant cells, confirming a hemorrhagic effusion. Surgical interventions, such as thoracotomy, decortication, or arterial embolization, may be required in refractory cases. However, given the poor prognosis of AML patients with hemothorax, treatment must be individualized, balancing aggressive intervention with overall prognosis and quality-of-life considerations [3,10]. Further studies are needed to refine management strategies and establish evidence-based guidelines [3,11]. The presentation of AML is often non-specific, with patients presenting symptoms such as fever, general malaise, infections, and hemorrhagic syndromes. More specific signs, such as anemia, thrombocytopenia, bone pain, or leukostasis, may be observed, as seen in our patient's complete blood count, but are not always present. Laboratory findings, including peripheral blood smears and bone marrow examination, are critical for diagnosis, with confirmation made through the identification of myeloid blasts [11-13]. AML is diagnosed through peripheral blood smears and bone marrow examination, with Auer rods or high leukocyte counts confirming the diagnosis. In our case, the bone marrow biopsy confirmed acute myelomonocytic leukemia (AML M4, FAB classification) with myelodysplasia-related changes (WHO classification). In the absence of these findings, additional tools like a leukoerythroblastic blood picture or dry tap may be needed. Patients often present with non-specific symptoms, delaying early diagnosis [10-13]. In addition to hemothorax, our patient suffered from spontaneous hemopericardium, a rare but severe complication of AML. Large-volume hemopericardium exacerbates hemodynamic instability and can progress to cardiac tamponade, significantly worsening the prognosis. The pathophysiology involves similar mechanisms to hemothorax, including thrombocytopenia, coagulopathy, and fragile neovascularization. Diagnosis requires urgent echocardiography, and management includes pericardiocentesis for hemodynamic stabilization and correction of underlying coagulopathy. As was the case in our patient, pericardiocentesis improved circulatory status, but subsequent septic shock and multiorgan failure ultimately led to a poor outcome [12].

The updated 2022 classification systems for AML incorporate molecular analysis, refining diagnosis, and risk stratification. Differences between the International Consensus Classification and the revised WHO system present both challenges and opportunities. The European Leukemia Net 2022 risk classification includes molecular data and MRD monitoring, although the use of minimal residual disease in treatment decisions remains controversial. AML is associated with hemostatic abnormalities, particularly thrombocytopenia and acquired von Willebrand syndrome (AVWS), due to leukemic bone marrow infiltration, which impairs platelet production and hemostasis [10,12,14]. AVWS results from the degradation of ultra-large von Willebrand factor (VWF) multimers, which impair platelet adhesion and aggregation. Additionally, hyperfibrinolysis, a breakdown of fibrin clots, further exacerbates the bleeding risk, often worsened by chemotherapy [15,16]. AML is a genetically complex disease with key gene alterations, including FLT3, NPM1, DNMT3A, IDH1, IDH2, TET2, RUNX1, NRAS, and TP53. The incidence of these mutations varies by age, prior hematologic conditions, and exposure to chemotherapy or radiotherapy. Since 2010, molecular data have shaped AML prognosis and treatment, integrating targeted therapies alongside traditional chemotherapy. The approval of midostaurin (FLT3 inhibitor) in 2017 marked a turning point, followed by additional targeted therapies: gilteritinib, quizartinib (FLT3); ivosidenib, olutasidenib (IDH1); and enasidenib (IDH2). The combination of hypomethylating agents with venetoclax has significantly improved outcomes in older adults. AML therapy is now personalized based on genomic profiling and patient comorbidities, optimizing treatment selection and hematopoietic cell transplant decisions in the era of precision medicine [17]. The introduction of all-trans retinoic acid (ATRA) and arsenic trioxide (ATO) has made acute promyelocytic leukemia (APL) the most curable subtype of AML. However, early mortality remains a significant challenge, primarily due to hemorrhagic complications, as well as infection and differentiation syndrome. APL is associated with DIC and hyperfibrinolysis, leading to life-threatening hemorrhages and thrombosis. While hemorrhage is the leading cause of death, thrombosis also contributes to morbidity and mortality. Despite advances in pathogenesis, risk prediction, and management, preventing early fatal complications remains difficult [18].

The prognosis of AML patients with hemothorax and hemopericardium remains poor due to the malignant nature of the disease and associated hemorrhagic complications [10]. While chemotherapy targets the leukemic burden, it does not directly resolve hemorrhagic events and may exacerbate coagulopathy [6]. Given the critical need for timely intervention, strategies such as platelet transfusions, tailored coagulation management, and surgical drainage can improve outcomes in selected patients, as was the case with our patient. However, due to the rarity of hemothorax and hemopericardium as presenting signs of AML, evidence-based management guidelines remain limited. Further studies are essential to optimize treatment strategies and establish clearer therapeutic approaches [6,7].

## Conclusions

Hemothorax is a rare but life-threatening complication of AML that requires immediate recognition and intervention. Its underreported nature and lack of clear guidelines make diagnosis challenging, particularly in emergency settings. Early diagnostic tools like ultrasound or chest X-ray are crucial for prompt detection and intervention. Spontaneous hemopericardium, as observed in our patient, is another severe AML complication that significantly impacts prognosis. "Early recognition through echocardiography and timely management with pericardiocentesis and coagulation correction are essential to prevent hemodynamic collapse. A multidisciplinary approach with hematologists, intensivists, and thoracic specialists is crucial. Balancing hemorrhagic control with leukemia treatment is key, with tailored interventions including coagulation support and thoracic procedures. This case highlights the need for clinical awareness, rapid diagnostics, and standardized guidelines to improve outcomes.
